# Causality analysis of fatty acids, triglycerides and psoriasis based on Mendelian randomization

**DOI:** 10.1097/MD.0000000000048422

**Published:** 2026-05-15

**Authors:** Jia Long, Caidi Deng, Manni Huang, Lulu Tang, Qiuju Li, Fuxi Wang, Youkun Lin

**Affiliations:** a Guangxi Medical University, Nanning, Guangxi, China; b Department of Dermatology and Venereology, Shenzhen Second People’s Hospital/The First Affiliated Hospital of Shenzhen University, Shenzhen, Guangdong, China; c Department of Dermatology and Venereology, The First Affiliated Hospital of Guang-xi Medical University, Nanning, Guangxi, China.

**Keywords:** Fatty acids, Mendelian randomization, psoriasis, single nucleotide polymorphisms, triglycerides

## Abstract

Studies have shown that an increase in the abundance of triglycerides (TG), oleic acid, and stearic acid can exacerbate the symptoms of psoriasis. Based on this, the present study aimed to employ Mendelian randomization (MR) methods to investigate the causal relationship between fatty acid (FA) and TG levels and the risk of psoriasis. A 2-sample MR analysis was performed using summary statistics from the genome-wide association studies of FA, TG, and psoriasis of European ancestry. Single nucleotide polymorphisms were used as instrumental variables in MR analysis. Inverse variance weighted was used as a primary method to study the causal relationship of FA on psoriasis. Similarly, inverse variance weighted was used to test for the causal effect of TG on psoriasis as well as the causal effect of FA on TG. Finally, sensitivity analysis was used to test the reliability of the MR analysis results. In this study, no significant association was observed between FA and psoriasis, indicating that FA was not a causal factor for psoriasis. TG had a causal relationship with psoriasis, and it was a risk factor for psoriasis. In MR analysis, it was demonstrated that FA did not directly exacerbate psoriasis; TG was a risk factor for psoriasis exacerbation. Our study elucidates the relationship between TG and FAs in psoriasis pathogenesis, thereby refining the understanding of its underlying mechanisms and offering novel theoretical and therapeutic avenues for diagnosis and treatment .

## 1. Introduction

Mendelian randomization (MR) is fundamentally an instrumental variable method derived from econometrics. MR can be used to test causal hypotheses. This is a genetic tool variable analysis that assesses potential relationships between exposure and outcomes.^[1]^ Because MR uses instrumental variable analysis to simulate the randomization process of causal reasoning in randomized controlled trials, the design is less susceptible to confounding and reverse causal bias.^[2]^

Psoriasis is a common autoimmune disease characterized by skin erythema, desquamation and joint pain, which can affect the physical and mental health of patients.^[3]^ The pathogenesis of psoriasis is not clear at present, but it is related to many factors such as heredity, environmental factors and autoimmunity.^[4]^ Recent studies have also found that psoriasis is related to intestinal flora disorders, host immunity and other diseases.^[5]^

Triglycerides (TG) are an organic compound that is produced by esterifying 3 hydroxyl groups of glycerol with 3 fatty acid (FA) molecules.^[6]^ The synthesis and breakdown of TG in adipose tissue and muscle is a key factor in energy metabolism, as it ensures that there is enough fuel available during starvation. Triglyceride turnover determines the availability of FAs for use by mammalian tissues.^[7]^ FAs are an important component of lipids (fat-soluble components of living cells) in plants, animals and microorganisms.^[8]^ The most widely distributed FAs are 16-carbon and 18-carbon FAs, called palmitic acid and stearic acid, respectively.^[9]^ Both palmitic acid and stearic acid are present in the lipids of most living organisms. In animals, palmitic acid makes up 30% of body fat. It makes up 5 to 50% of the lipids in plant fat.^[10,11]^

People with psoriasis are more likely to have obesity, dyslipidemia, atherosclerosis, and nonalcoholic fatty liver disease.^[12]^ It has also been suggested that an increase in the abundance of oleic acid and stearic acid intestinal can exacerbate the psoriasis symptoms.^[5]^ It has been reported that lipid metabolism disorders are closely related to the occurrence and development of psoriasis.^[13,14]^ Therefore, based on MR method, this study takes FAs and TG as exposure factors, and takes TG and psoriasis as outcomes, providing a new reference for the causal relationship between FAs, TG and psoriasis.

## 2. Materials and methods

### 2.1. Data source and IVs selection

Exposure factors and outcome datasets were obtained from integrative epidemiology unit (IEU) OpenGWAS (https://gwas.mrcieu.ac.uk/).^[15]^ The genome-wide association statistics identity of FA was met-d-Total_FA (114,999 samples contained 12,321,875 single nucleotide polymorphisms [SNPs]), TG was ieu-b-111 (441,016 samples contained 12,321,875 SNPs) and psoriasis was finn-b-L12_PSORIASIS (216,752 samples contained 16,380,464 SNPs). Moreover, for FA, TG and psoriasis, these genome-wide significant SNPs (*P* < 5*10^-8^) were used as IVs. They were tested for independent inheritance (*R*^2^ = 0.001) without linkage disequilibrium among themselves. This study also estimated *R*^2^ for the exposure variance interpreted by each IV.^[16]^

### 2.2. MR analysis

In this study, “TwoSampleMR” R package (version 0.5.6, MR IEU, University of Bristol, Bristol, UK)^[17]^ was used for a 2-sample MR analysis between exposure factors (FA and TG) and outcomes (psoriasis and TG), 5 common MR methods were applied: MR-Egger regression,^[18]^ inverse variance weighted (IVW) method,^[19]^ weighted median test,^[20]^ weighted mode test^[21]^ and simple mode test^[22]^ were used for features that contained more than 1 IV. The IVW test was primary method. If the assumption that all included SNPs are valid IV was satisfied, the IVW method provided an accurate estimate.^[23]^ The other methods were used as supplementary analysis methods.

### 2.3. Sensitivity analysis

In this study, sensitivity analysis was used to verify the reliability of MR analysis results. First, IVW and MR-Egger regression were used to test the heterogeneity of SNPs.^[24]^ If Q pval was < .05, it indicated that there was heterogeneity in SNPs. Afterwards, in order to evaluate whether there were confounding factors in this study, horizontal pleiotropic analysis^[25]^ was performed by MR-Egger regression. If *P* > .05 indicated that there were no confounding factors. Lastly, the leave-one-out (LOO) method was used to detect whether the remaining SNPs had an impact on the overall analysis, even if 1 SNP was removed.^[26]^

## 3. Results

### 3.1. The causal effect of FA on psoriasis

In this study, a total of 64 SNPs were obtained which associated with FA. The IVW method showed that no significant association of FA with psoriasis (*P* = .1631) (Table 1). There were no indication of causal link between FA and psoriasis, FA was also not the cause of psoriasis.

**Table 1:** MR analysis results for FAs, TG and psoriasis.

Outcome	Exposure	Method	nSNP	B	SE	*P* value	OR
Psoriasis ‖ id:finn-b-L12_PSORIASIS	Total FA ‖ id:met-d-Total_FA	MR Egger	60	0.293235876	0.132703226	.031086538	1.340759005
Psoriasis ‖ id:finn-b-L12_PSORIASIS	Total FA ‖ id:met-d-Total_FA	Weighted median	60	0.103644984	0.102736501	.313050008	1.109206599
Psoriasis ‖ id:finn-b-L12_PSORIASIS	Total FA ‖ id:met-d-Total_FA	Inverse variance weighted (fixed effects)	60	0.093495104	0.067029721	163066937	1.098005227
Psoriasis ‖ id:finn-b-L12_PSORIASIS	Total FA ‖ id:met-d-Total_FA	Simple mode	60	0.193131263	0.17725753	.280340867	1.213042011
Psoriasis ‖ id:finn-b-L12_PSORIASIS	Total FA ‖ id:met-d-Total_FA	Weighted mode	60	0.124363832	0.096757355	.203704248	1.13242781
Psoriasis ‖ id:finn-b-L12_PSORIASIS	TG ‖ id:ieu-b-111	MR Egger	284	0.086729054	0.095528104	.364711289	1.090601145
Psoriasis ‖ id:finn-b-L12_PSORIASIS	TG ‖ id:ieu-b-111	Weighted median	284	0.182937288	0.089908153	.04187976	1.200739105
Psoriasis ‖ id:finn-b-L12_PSORIASIS	TG ‖ id:ieu-b-111	Inverse variance weighted (fixed effects)	284	0.152785583	0.053729931	.004460922	1.16507514
Psoriasis ‖ id:finn-b-L12_PSORIASIS	TG ‖ id:ieu-b-111	Simple mode	284	0.267348026	0.179704112	.137940134	1.306495061
Psoriasis ‖ id:finn-b-L12_PSORIASIS	TG ‖ id:ieu-b-111	Weighted mode	284	0.17664605	0.084282769	.036981579	1.193208682

B = beta coefficient, FA = fatty acid, nSNP = number of single nucleotide polymorphism, OR = odds ratio, SE = standard error, TG = triglyceride.

### 3.2. The causal effect of TG on psoriasis

**Figure 1.**
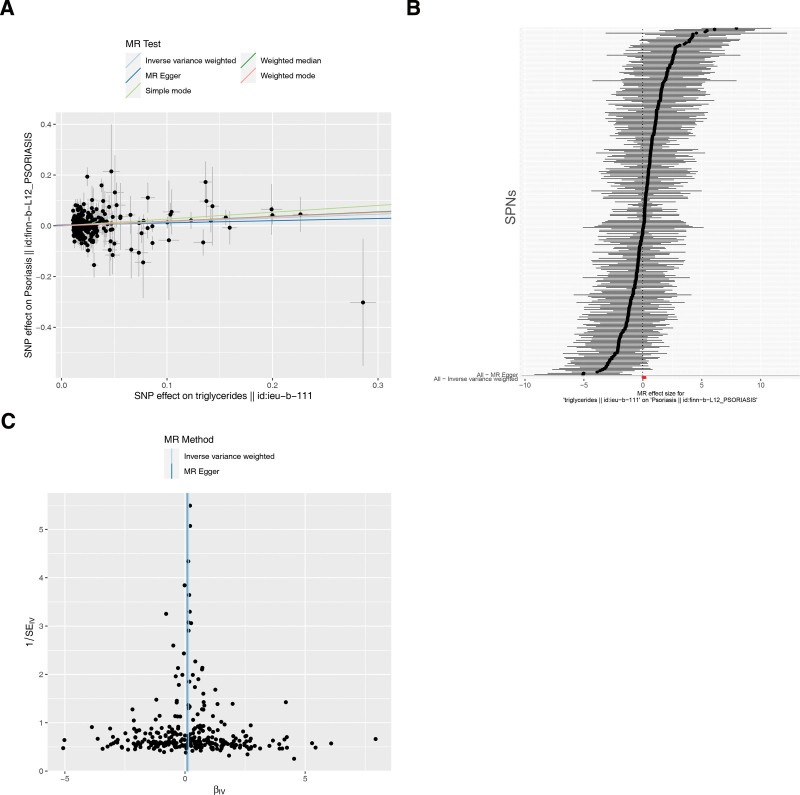
The relationship between TG and psoriasis was based on MR analysis. (A) Scatter plots were used to determine the association between exposure factors and outcomes. The abscissa is the effect of SNP on exposure, the ordinate is the effect of SNP on outcome, and the colored line represents the fitting results of different MR Algorithms. A regular slope of the line represents a risk factor, and a negative slope represents a safety factor. (B) The diagnostic efficacy of each SNP site predicted exposure factor on the outcome was judged by the forest plot, SNP points on the left were decreased (safety factor), and SNP points on the right were increased (risk factor). (C) Random determination of MR According to Mendelian second law randomization by funnel plot. MR = Mendelian randomization, SE = standard error, SNP = single nucleotide polymorphism, TG = triglycerides.

**Figure 2.**
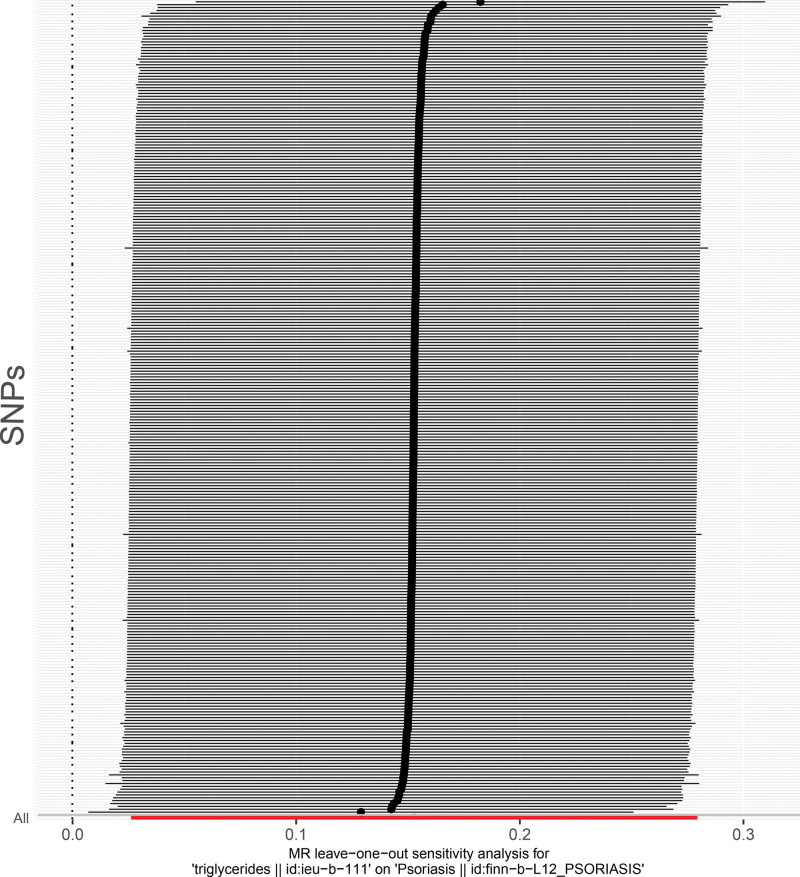
Sensitivity analysis was used to determine the reliability of the results. MR = Mendelian randomization, SNP = single nucleotide polymorphism.

A total of 296 SNPs independently associated with TG were obtained. The IVW results showed that TG had a causal relationship on psoriasis (*P* = .0045) (Table 1), it was a risk factor for psoriasis (odds ratio = 1.1651). The slope of the IVW result was positive and the intercept was little (Fig.1A). IVW results showed that SNPs were > 0, which suggested that TG was a risk factor for psoriasis (Fig.1B). The funnel diagram showed that SNPs were randomly distributed on both sides of the IVW line, indicating that it conformed to Mendel second law (Fig.1C). Afterwards, sensitivity analysis proved MR analysis results were valid. Firstly, the heterogeneity test showed that despite the heterogeneity in SNPs, the *P* value of IVW method was < .05, so there was no impact on the results. Then, the *P* value of the horizontal pleiotropy test was .3486, indicating the absence of confounding factors. Finally, the results of LOO analysis showed that by gradually eliminating each SNP, the effect of the remaining SNPs on the outcome variables did not change much (Fig.2). To sum up, the MR analysis in this study was reliable and stable.

### 3.3. The causal effect of FA on TG

**Figure 3.**
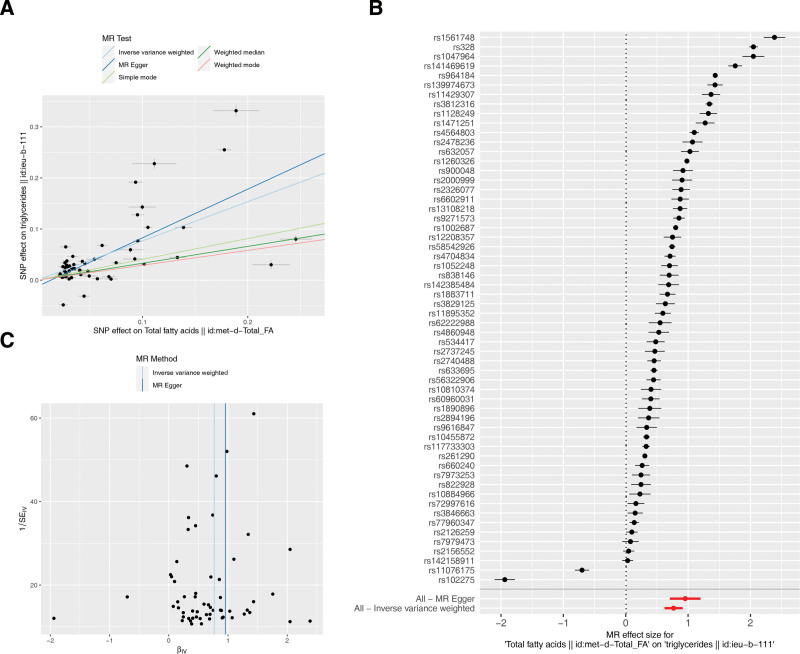
The relationship between FA and psoriasis was based on MR analysis. (A) Scatter plot was used to determine the correlation between exposure factors and outcomes. The abscissa was the effect of SNP on exposure, the ordinate was the effect of SNP on outcomes, and the colored line represented the fitting results of different MR Algorithms. A regular slope represented a risk factor, and a negative slope represented a safety factor. (B) The forest plot was used to judge the diagnostic efficacy of each SNP site in the prediction of exposure factors for the outcome, SNP points on the left side were decreased (safety factor), SNP points on the right side were increased (risk factor); The overall effect of the bottom 2 behaviors in different models can also be seen as the Inverse variance weighted method also illustrates FA as risk factors for TG. (C) Random determination of MR According to Mendelian second law randomization by funnel plot. FA = fatty acid, MR = Mendelian randomization, SE = standard error, SNP = single nucleotide polymorphism, TG = triglycerides.

**Figure 4.**
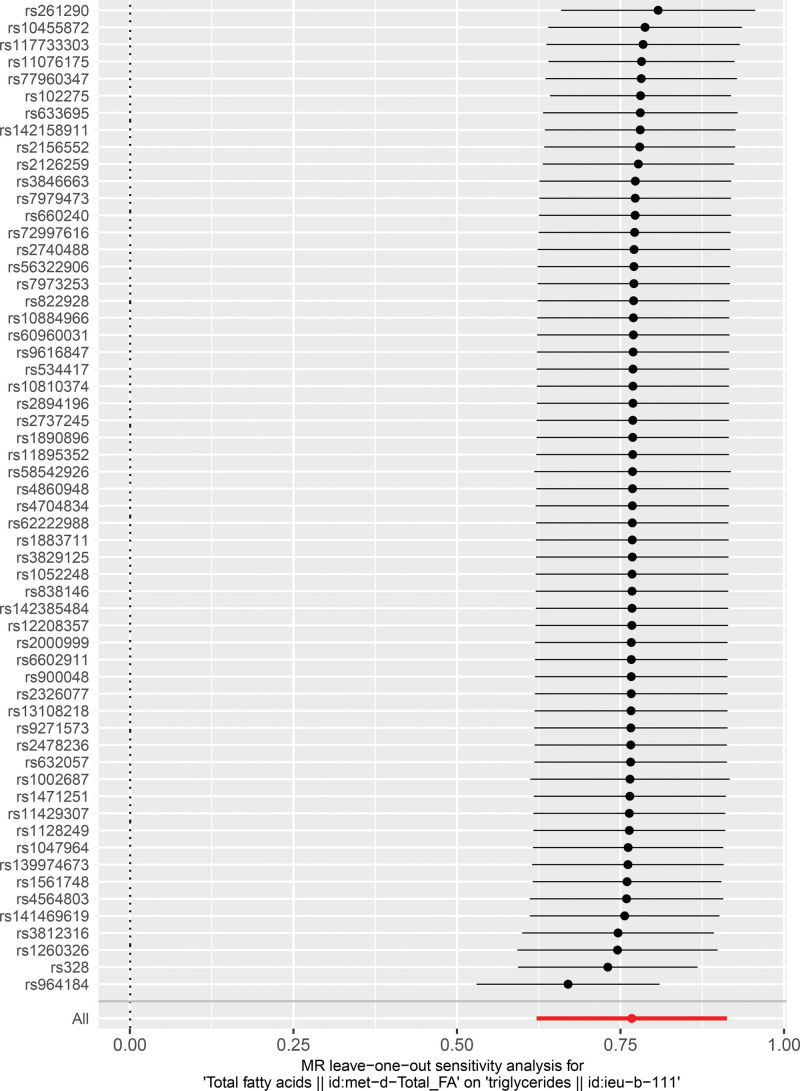
Sensitivity analysis was used to determine the reliability of the results. FA = fatty acid, MR = Mendelian randomization.

A total of 296 SNPs were used as fatty-TG MR analysis. Based on IVW method, FA had a causal relationship on TG (*P* < .001) (Table 1). The scatter plot revealed that the slope of the IVW analysis was positive (Fig.3A). Afterwards, the SNPs of IVW analysis and MR-Egger regression results were both > 0 (Fig.3B). In summary, FA was a risk factor for TG. Meanwhile, MR analysis conformed to Mendel second law of random grouping (Fig.3C). Similarly, sensitivity analysis proved the reliability of MR analysis results. There were no confounding factors in the horizontal pleiotropy test (*P* = .0726), and there were no outliers for SNPs by the LOO method (Fig.4). In conclusion, TG was associated with psoriasis, they were the risk factors for exacerbating psoriasis, FA had a causal effect on TG, however, no significant causal relationship between FA and psoriasis, and FA did not directly aggravate psoriasis.

## 4. Discussion

This study was based on the IEU OpenGWAS database and employed MR to analyze the causal relationships among 3 exposure factors: psoriasis, FAs, and TG. Further causal inference revealed that the exposure factor FAs does not directly exacerbate or cause psoriasis, whereas TG are the risk factor that exacerbates/causes psoriasis. Through the above analysis, the study investigated the influence mechanisms of FAs and TG on psoriasis, providing a theoretical basis and reference value for psoriasis research, which indicates that FAs are a risk factor for increased TG, while TG are a risk factor exacerbating/causing psoriasis.

Psoriasis as a skin disease with unclear pathogenesis and various pathogenic factors, it is necessary to find and improve the markers conducive to the diagnosis and treatment of psoriasis.^[3,27]^ As an important substance of energy metabolism in human body, triglyceride can not only supply energy for the body, but also promote the utilization of FAs.^[6]^ At the same time, FAs as the main component of lipids in the body have been proved to promote the occurrence and development of a variety of diseases.^[28]^ For example, Wenxing Su et al found that the increased levels of total cholesterol, triglyceride, low-density lipoprotein and ultra-low-density lipoprotein in patients with psoriasis, accompanied by increased low-density granulocyte infiltration, may be risk factors for atherosclerosis in patients with psoriasis.^[29]^ In addition, during psoriatic inflammation, patients exhibit reduced levels of lipoprotein, leptin, and mucin, alongside insulin resistance, which further exacerbates psoriasis.^[30,31]^ Kaur et al demonstrated that obese psoriasis patients had increased systemic inflammation and oxidative stress, evidenced by glutathione redox ratio, compared to normal-weight patients.^[32]^ Carrascosa et al noted that obesity increases the risk of adverse effects of systemic therapy for psoriasis and may lead to increased dosage of biologics to avoid ineffective reductions of these drugs.^[33]^ All in all, TG and FAs are not only a kind of human energy substances involved in the normal metabolism of the body, their imbalance and disorder seem to make some diseases, including psoriasis, easier to occur.^[5]^

In this study, we investigated the roles of TG and FAs in the pathogenesis of psoriasis. Notably, this study is also the first time to use MR to analyze the causal relationship among them. Previous studies have shown that the imbalance of intestinal microflora will accelerate host lipid metabolism disorders and the development of psoriasis, while patients with psoriasis are more likely to develop diseases related to lipid metabolism disorders.^[5,12]^ In this study, we used heterogeneity test, multiplicity test and MR analysis to find that FA does not directly aggravate psoriasis, and TG is a risk factor for the aggravation of psoriasis. In the present study, triglyceride levels were positively correlated with psoriasis severity. And although FAs do not influence the course of psoriasis as a direct factor, the possible involvement of FAs in the progression of psoriasis as an indirect contributors is still of interest.

In addition, the knowledge of how TG and FAs affect inflammatory factors and cells during the development of psoriasis is also still relatively scarce and needs to be further explored. Of course, larger genome-wide association study summary data and more updated MR analysis of genetic variants are necessary to further verify the causal relationship between TG, FAs and psoriasis, and we will continue to pay attention to the analysis of psoriasis, FAs and TG. For this result, it may also be necessary to analyze the FA composition of patients with psoriasis and normal subjects. At the same time, the actual nutritional status of patients with psoriasis needs to be taken into account when evaluating the role of fat metabolism in the pathogenesis of psoriasis. In short, our study revealed the relationship between TG and FAs in the occurrence and development of psoriasis, further improved and clarified the pathogenic mechanism of psoriasis, and provided new theoretical insights and therapeutic opportunities for the diagnosis and treatment of psoriasis.

## Author contributions

**Conceptualization:** Fuxi Wang, Youkun Lin.

**Data curation:** Manni Huang.

**Formal analysis:** Qiuju Li.

**Investigation:** Caidi Deng.

**Validation:** Lulu Tang.

**Writing – original draft:** Jia Long.

**Writing – review & editing:** Fuxi Wang, Youkun Lin.
